# Research progress in hepatitis B virus covalently closed circular DNA

**DOI:** 10.20892/j.issn.2095-3941.2021.0454

**Published:** 2021-12-22

**Authors:** Xiaodong Zhang, Yufei Wang, Guang Yang

**Affiliations:** 1 Department of Gastrointestinal Cancer Biology, Liver Cancer Center, Tianjin Medical University Cancer Institute & Hospital, National Clinical Research Center for Cancer, Key Laboratory of Cancer Prevention and Therapy, Tianjin, Tianjin’s Clinical Research Center for Cancer, Tianjin 300060, China; 2 Department of Cancer Research, College of Life Sciences, Nankai University, Tianjin 300071, China

**Keywords:** Hepatitis B virus, cccDNA, HBx, hepatocarcinogenesis, epigenetic modulation, therapy

## Abstract

Hepatitis B virus (HBV) infections are a global public health issue. HBV covalently closed circular DNA (cccDNA), the template for the transcription of viral RNAs, is a key factor in the HBV replication cycle. Notably, many host factors involved in HBV cccDNA epigenetic modulation promote the development of hepatocellular carcinoma (HCC). The HBV cccDNA minichromosome is a clinical obstacle that cannot be efficiently eliminated. In this review, we provide an update on the advances in research on HBV cccDNA and further discuss factors affecting the modulation of HBV cccDNA. Hepatitis B virus X protein (HBx) contributes to HBV cccDNA transcription and the development of hepatocarcinogenesis through modulating host epigenetic regulatory factors, thus linking the cccDNA to hepatocarcinogenesis. The measurable serological biomarkers of continued transcription of cccDNA, the effects of anti-HBV drugs on cccDNA, and potential therapeutic strategies targeting cccDNA are discussed in detail. Thus, this review describes new insights into HBV cccDNA mechanisms and therapeutic strategies for cleaning cccDNA, which will benefit patients with liver diseases.

## Introduction

Chronic infection with hepatitis B virus (HBV) remains a major public health issue. Globally, more than 296 million people were chronically infected with HBV in 2019 and were therefore at risk of end-stage liver disease and hepatocellular carcinoma (HCC)^1–3^. HBV covalently closed circular DNA (cccDNA), the template for the transcription of viral RNAs, is a key factor in the HBV replication cycle^4,5^. In the HBV life cycle, HBV enters hepatocytes by interacting with NTCP; this is followed by uncoating and transport of the relaxed circular DNA (rcDNA) into the nucleus. As an intermediate, the cccDNA minichromosome is formed in the host cell nucleus from the rcDNA genome, which is associated with histone and non-histone proteins. The viral proteins hepatitis B core antigen (HBcAg), hepatitis B e antigen (HBeAg), HBV polymerase (pol), hepatitis B surface antigen (HBsAg), and HBV X protein (HBx) are produced from the cccDNA. The pgRNA transcribed from the cccDNA is selectively packaged inside core particles. These mature core particles can be enveloped for release as virions or transported to the nucleus to generate more cccDNA. Thus, cccDNA provides the molecular basis for establishing and maintaining viral infection^6–8^.

The development of HCC is affected by interactions among genetic predisposition, environmental factors, and viruses. Chronic inflammation, epigenetic modifications, DNA damage, senescence, chromosomal instability, and early neoangiogenesis drive the development and progression of HCC^9,10^. The risk of HCC is also correlated with HBV’s replication, genotype, and genomic mutations^11–14^. The HBV-integrated host genome directly results in the development of HCC^15–17^. In addition, many other viral and host factors, such as mTOR, contribute to the development of HCC^18–20^. The key molecular basis of HBV persistence involves cccDNA, which plays a crucial role in the development of HCC^3^. The HBV cccDNA minichromosome is maintained throughout the clinical phases of chronic hepatitis. Because of cccDNA’s key role and function in the viral replication cycle, clinical reports have indicated that measuring and eliminating cccDNA are important^6,21^.

Given the significance of cccDNA, this review discusses research progress including the composition, formation, maintenance, regulation, and epigenetic modulation of the cccDNA minichromosome; the relationships between HBx-mediated cccDNA minichromosome and HCC; detection of HBV cccDNA; anti-HBV drugs targeting cccDNA; and possible curative strategies aimed at eliminating or hindering the viral cccDNA.

## Mapping the HBV cccDNA minichromosome

In the nucleus, HBV cccDNA is formed from rcDNA; bound to both histones (H2A, H2B, H3, H4, and H1) and non-histone proteins (HBc, HBx, and host factors); and organized into a chromatin-like structure termed the HBV cccDNA minichromosome, which shows a typical “beads-on-a-string” arrangement under electron microscopy^22,23^. Histone proteins H2A, H2B, H3, H4, and H1 are detectable by immunoblotting on purified nucleoprotein complexes^24^. The HBV core protein (HBc) is a component of the HBV minichromosome. HBc binds the cccDNA minichromosome *in vivo* and *in vitro*, thus decreasing the nucleosomal spacing of the HBV cccDNA^24^.

Moreover, HBx in association with the cccDNA minichromosome, initiates and maintains HBV replication. HBx has been shown to activate HBV transcription through its recruitment to cccDNA and to consequently increase the recruitment of co-activators such as CBP/p300 and PCAF, which in turn target the promoters and activate gene expression, partly through histone acetylation^25–28^. Furthermore, HBx binds the parvulin 14 (Par14) and parvulin 17 (Par17) proteins, and recruits Par14/Par17 to the cccDNA minichromosome, thereby promoting the transcriptional activation of cccDNA^29^. Pre-mRNA processing factor 31 (PRPF31) is recruited to cccDNA through interacting with HBx in the nucleus, as discovered through chromatin immunoprecipitation assays^30^. Interferon-inducible protein 16 (IFI16), structural maintenance of chromosomes (SMC) complex 5/6, HP1, SMCHD1, and PML bind HBV cccDNA in hepatic nuclei, and are associated with suppression of cccDNA transcription^31–34^. APOBEC3A binds cccDNA through interacting with the HBc protein^1^. Furthermore, cellular transcription factors such as CREB, ATF, YY1, STAT1, and STAT2, and chromatin modification enzymes such as GCN5, HDAC1, SIRT1, PRMT1, PRMT5, EZH2, and SETDB1 have been shown to be associated with cccDNA through chromatin immunoprecipitation assays^25,35–37^. The lncRNA DLEU2 and HBx are co-recruited to cccDNA and subsequently play a role in the regulation of HBV^37^. Our group has reported that HAT1 confers the assembly of the cccDNA minichromosome^38^. PCNA participates in the structural organization of the cccDNA minichromosome^39^. HAT1 silencing decreases the deposition of HBx and p300 onto the cccDNA minichromosome in HBV-infected dHepaRG and HepG2-NTCP cells. HAT1 anchors to the cccDNA minichromosome through interaction with HBc, while HULC serves as a scaffold in the complex of HAT1/HULC/HBc, thereby modulating the acetylation of histones on the cccDNA minichromosome^25,38,40^.

The mechanisms permitting the conversion from rcDNA to cccDNA remain largely unknown. Assembly of a variety of viral and host factors, including HBc, HBx, histones, and non-histone proteins, on cccDNA is key in the formation and maintenance of the cccDNA minichromosome. The HBc carboxyl-terminal domain contains the nuclear localization signal, which plays an important role in cccDNA minichromosome formation *via* delivering the rcDNA into the nucleus from mature nucleocapsids^41^. Mutations in the HBc N-terminal domain increase cccDNA minichromosome formation through controlling the release of rcDNA from mature capsids and the nuclear import of rcDNA^42,43^. The host ATR-CHK1 pathway is involved in cccDNA minichromosome formation through processing HBV rcDNA conversion to cccDNA^44^. In addition, SAMHD1, a component of the innate immune system that regulates the deoxyribonucleoside triphosphate levels required for host and viral DNA synthesis, has a role in regulating cccDNA minichromosome formation^45^. PRPF31 may enhance cccDNA minichromosome formation or maintenance by interacting with HBx in the nucleus^30^. Cellular DNA topoisomerase I (TOP1) and II (TOP2) are involved in catalyzing both *de novo* synthesis and in intracellular amplification of the cccDNA minichromosome^46^. Furthermore, several host cellular DNA repair proteins, such as tyrosyl-DNA phosphodiesterase 2 (TDP2), DNA polymerase (Pol), flap endonuclease 1 (FEN1), and DNA ligases, are required for cccDNA synthesis in *de novo* infection and intracellular amplification^2,47–49^. Five core components of lagging-strand synthesis have been identified and defined as the minimal set of factors essential for cccDNA minichromosome formation: proliferating cell nuclear antigen, the replication factor C complex, DNA polymerase δ, flap endonuclease 1, and DNA ligase 1^50,51^.

**Figure 1:**
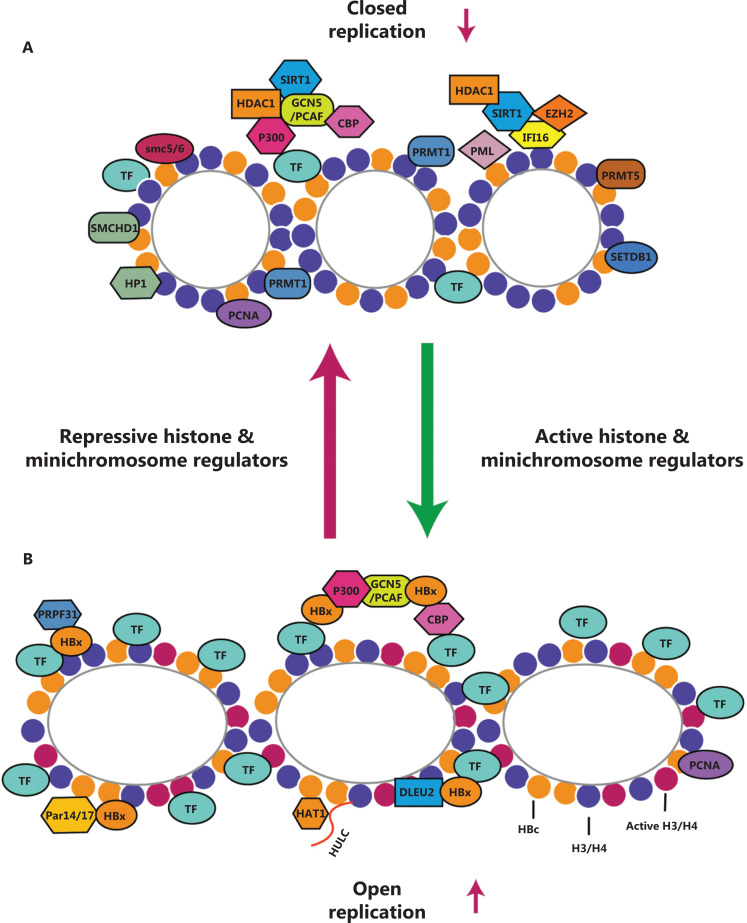
Composition of the HBV cccDNA minichromosome. In the nucleus, HBV cccDNA is converted from rcDNA, and both histones and non-histone proteins are attached. (A) Various factors directly or indirectly binding cccDNA, such as HBx, HAT1, and p300, promote the replication of HBV. (B) Some restriction factors, such as SIRT1, HDAC1, PRMT1/5, and IFI16, are loaded on cccDNA, thus inhibiting HBV replication. A3A: APOBEC3A. TF: transcription factors, such as CREB, ATF, YY1, STAT1, and STAT2.

With respect to the roles of factors in cccDNA minichromosome maintenance, current evidence suggests that APOBEC3A and APOBEC3B anchor on the cccDNA minichromosome by interacting with HBc, thus resulting in the degradation of HBV cccDNA^1,3^. The baseline levels of APOBEC3A and APOBEC3B significantly limit the formation and accumulation of the cccDNA minichromosome^52^. High levels of ubiquitin conjugating enzyme E2 L3 (UBE2L3) maintain cccDNA stability by inducing the degradation of APOBEC3A^53^. Our group has reported that MSL2 and HULC maintain HBV cccDNA minichromosome stability through the degradation of APOBEC3B in hepatoma cells^54–56^. Furthermore, myxovirus resistance 2 (MX2) protein, an interferon-α (IFN-α) inducible effector, inhibits HBV infection by decreasing the levels of cccDNA, probably through indirectly impairing the conversion of rcDNA to cccDNA rather than destabilizing existing cccDNA^57^. The above findings are summarized in **Figure 1** and **Table 1**.

**Table 1:** Summary of factors on HBV cccDNA and their functions

Name	Function	Reference
HBx	Prevents the recruitment of transcriptional repressors or recruits transcription factors to cccDNA, thus activating transcription of HBV genes *via* epigenetic regulation; degrades SMC5/6, which binds cccDNA and inhibits its transcription	^31^
HBc	Enhances the formation of cccDNA *via* delivering the rcDNA to the nucleus from mature nucleocapsids	^41^
CBP/p300	Enhances the acetylation of cccDNA-bound histones	^25^
PCAF/GCN5	Enhances the acetylation of cccDNA-bound histones	^25^
ATF	Enhances cccDNA transcription	^25^
CREB	Enhances cccDNA transcription	^25^
YY1	Enhances cccDNA transcription	^25,35^
STAT1/2	Enhances cccDNA transcription	^25,35^
Par14/17	Enhances cccDNA transcription activation by binding HBx	^29^
PRPF31	Enhances cccDNA minichromosome formation or maintenance by interacting with HBx	^30^
HDAC1	Inhibits cccDNA transcription as a transcriptional suppressor	^34^
SIRT1	Inhibits cccDNA transcription as a chromatin modification enzyme	^34^
IFI16	Inhibits cccDNA transcription through enhancing recruitment of transcriptional suppressors and depressing transcriptional activators	^34^
SMCHD1	Suppresses cccDNA transcription	^34^
PML	Inhibits cccDNA transcription	^34^
SMC5/6	Inhibits cccDNA transcription	^31^
SETDB1	Inhibits transcription of cccDNA by SETDB1 histone methyltransferase	^32^
HP1	Inhibits cccDNA transcription	^32^
PRMT1/5	Mediates epigenetic suppression of the cccDNA minichromosome	^36^
EZH2	Inhibits cccDNA transcription	^34,37^
DLEU2	After co-recruitment to cccDNA with HBx, displaces EZH2 from the viral chromatin and boosts cccDNA transcription	^37^
HAT1	Modulates acetylation of histones on the cccDNA minichromosome	^38^
HULC	Serves as a scaffold in the complex of HAT1/HULC/HBc; modulates acetylation of histones on the cccDNA minichromosome	^38^
PCNA	Enhances cccDNA formation from rcDNA	^39^

HBx, hepatitis B X protein; HBc, hepatitis B core protein; CBP, CREB binding protein; PCAF, P300/CBP associated factors; GCN5, general control non-derepressible 5; ATF, artificial transcription factor; CREB, cAMP-response element binding protein; YY1, Yin Yang 1; STAT1/2, signal transducer and activator of transcription 1/2; Par14/17, parvulin 14/17; PRPF31, pre-messenger RNA processing factor 31; HDAC1, histone deacetylase 1; SIRT1, sirtuin 1; IFI16, interferon-inducible protein 16; SMCHD1, structural maintenance of chromosomes flexible hinge domain containing 1; PML, promyelocytic leukemia protein; SMC5/6, structural maintenance of chromosomes complex5/6; SETDB1, SET domain bifurcated histone lysine methyltransferase 1; HP1, heterochromatin-associated protein 1; PRMT1/5, protein arginine methyltransferase 1; EZH2, enhancer of zeste homolog 2; DLEU2, deleted in lymphocytic leukemia 2; HAT1, histone acetyltransferase 1; HULC, highly upregulated in liver cancer; PCNA, proliferating cell nuclear antigen.

## Regulation and epigenetic modulation of the HBV cccDNA minichromosome

### Viral factors on the cccDNA minichromosome

Regulation of the HBV cccDNA minichromosome is mediated by viral and host factors as well as inflammatory cytokines, through epigenetic modifications of cccDNA-bound histones^5,37,58^. As a viral protein, HBx, particularly HBx amino acid residues 55–60 and 121–126^28^, play key roles in stimulating the transcription of HBV cccDNA^31^. On the one hand, HBx enables cccDNA transcription by hijacking the cellular DDB1-containing E3 ubiquitin ligase, which degrades SMC5/6, a complex that binds the cccDNA minichromosome and inhibits its transcription^31^. This important mechanism provides the foundation through which HBx regulates cccDNA transcription. On the other hand, HBx prevents transcriptional repressor recruitment to the cccDNA minichromosome or recruits the transcription factors that activate the transcription of HBV genes *via* epigenetic regulation^25^. HBx regulates chromatin-mediated transcriptional repression of the cccDNA minichromosome through SETDB1 histone acetylation and methyl transfer, and the recruitment of heterochromatin protein 1 factor (HP1), which is correlated with condensed chromatin^32^. Similarly, HBx relieves SIRT3-mediated cccDNA transcriptional repression by inhibiting both SIRT3 expression and its recruitment to the cccDNA minichromosome^59^. In addition, HBx stimulates viral replication *via* DNA methylation of C-1619 in the cccDNA minichromosome^27^. Beyond viral chromatin, HBx recruits a variety of coding genes and non-coding RNA promoters associated with cccDNA, thus further regulating the cccDNA minichromosome^26,60,61^.

HBc may regulate cccDNA transcription through epigenetic modification. HBc preferentially binds CpG island 2 of the cccDNA minichromosome and alters the cccDNA minichromosome methylation profile, thus regulating the active transcription of cccDNA^62–64^. Furthermore, HBc carboxyl-terminal domain arginine residues in clusters III and IV may play an important role in the regulation of HBV transcription through decreasing the interaction of HBc with the cccDNA minichromosome and the acetylation of cccDNA-bound histones^65^. In addition, virion-delivered HBc stably associates with the integrated viral DNA and participates in early stages of cccDNA formation and/or transcription^66^. In contrast, a decreased amount of HBc protein on the cccDNA minichromosome does not account for the strong default of HBV RNAs in dHepaRG cells^32^.

### Host factors on the cccDNA minichromosome

Beyond viral proteins, host factors also play key roles in the regulation of cccDNA transcription. IFI16 is negatively correlated with HBV and serves as a unique innate sensor that recognizes and binds the HBV cccDNA minichromosome in hepatic nuclei, thereby inhibiting cccDNA transcription and HBV replication through enhancing the recruitment of transcriptional suppressors (HDAC1, SIRT1, or EZH2) and the inhibition of transcriptional activators (p300 or CBP) anchoring to the cccDNA minichromosome^34^. However, whether HBx mediates the expression of IFI16 remains unclear. Par 14 and Par 17, isoforms of the PPIase encoding PIN4 gene, bind HBx and the cccDNA minichromosome and up-regulate HBV transcription from cccDNA in an HBx-dependent manner^29^. Notch signaling facilitates cccDNA transcription *via* a cAMP response element-binding protein with E3 ubiquitin ligase modulation^67^. The LXR pathway with synthetic LXR agonists elicits potent anti-HBV activity in PHHs, possibly *via* sustained suppression of cccDNA transcription^68^. Histone deacetylase 11 (HDAC11) inhibits HBV transcription and replication in HBV-transfected Huh7 cells^69^. Both PRMT1 and PRMT5 also effectively restrict HBV transcription and replication, which mediate epigenetic suppression of the cccDNA minichromosome^36,70^. Neuronal precursor cell-expressed developmentally down-regulated protein 8 (NEDD8), a ubiquitin-like protein activating the ubiquitin-dependent proteasome pathway, is associated with the transcription of cccDNA. NEDD8-activating enzyme inhibitor is an efficient antiviral agent, which significantly restores SMC5/6 protein levels, and suppresses viral transcription and protein production in the HBV mini-circle system of HBV replication in both *in vitro* models and primary human hepatocytes infected naturally with HBV^71^. Factors such as HDM2 and ERK are associated with the activities of NEDD8 and may serve as potential targets for HBV therapy^72,73^. Host HAT1 is involved in modulating the acetylation of histones on the cccDNA minichromosome through interacting with HBc^38^.

In addition, miRNAs and lncRNAs contribute to the regulation of cccDNA transcription. The lncRNA DLEU2 and HBx are co-recruited to the cccDNA minichromosome, where they displace EZH2 from the viral chromatin, and boost transcription and viral replication^37^. Another lncRNA, Hox transcript antisense intergenic RNA (HOTAIR), is associated with the transcription of cccDNA. In HBV infection, HBx induces the down-regulation of DEAD box protein 5 (DDX5), thus resulting in the transcriptional reactivation of polycomb repressive complex 2 (PRC2)/HOTAIR target genes, including HBV cccDNA-encoded genes^74^. MiR-20a may be loaded onto AGO2 before its translocation into the nucleus, thus inducing methylation of the cccDNA minichromosome in human hepatoma cells, and leading to the suppression of HBV replication^75^. MiR-548ah promotes the replication and expression of HBV through regulating its target gene HDAC4. Inhibition of HDAC4 by miRNA-548ah may influence the deacetylation state of histones binding to the cccDNA minichromosome, thus leading to the replication of cccDNA^76,77^. HBV-infected HepG2-hNTCP-C4 cells and HBV transgenic mice treated with miR-302c-3p display decreased pgRNA and HBsAg mRNA concentrations as well as amounts of cccDNA^78,79^.

## HBx links the cccDNA minichromosome to hepatocarcinogenesis

**Figure 2:**
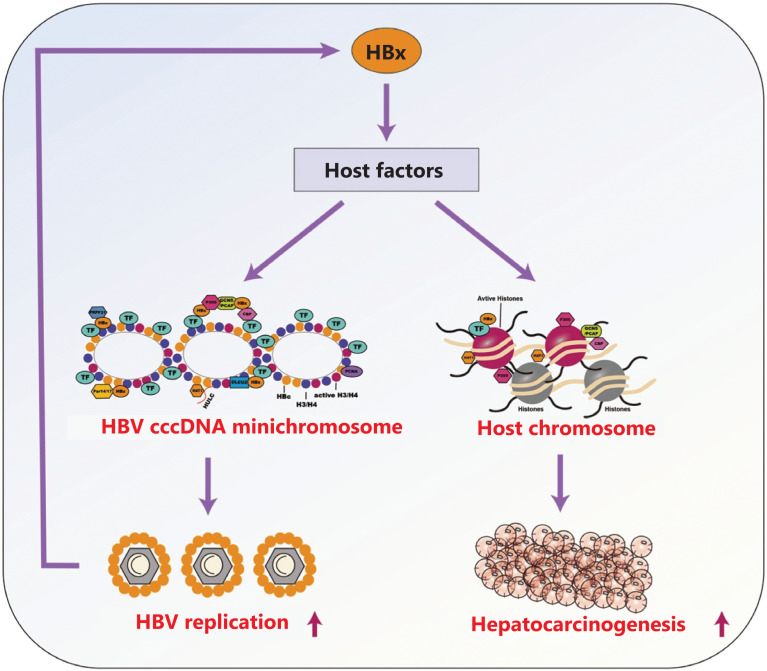
Hypothesis schematic of the roles of HBx-mediated epigenetic regulatory factors in the HBV cccDNA minichromosome and host chromosome. On the one hand, HBx up-regulates and recruits host epigenetic regulatory factors to cccDNA, thus enhancing the transcription of cccDNA through epigenetic regulation and leading to the replication of HBV in a positive feedback loop. On the other hand, HBx up-regulates host epigenetic regulatory factors, thus enhancing the growth of liver cancer, or directly results in the development of hepatocarcinogenesis.

Many factors that regulate the HBV cccDNA minichromosome are involved in the development of HCC, such as HBx. In virus-host interactions, several host factors determine the composition of the cccDNA minichromosome; host factors such as MSL2, DLEU2, HULC, and Notch signaling are regulated by HBx, thus forming a positive feedback loop^37,55,56,67,80^. Therefore, we presumed that HBx might enable HBV cccDNA transcription and the development of hepatocarcinogenesis through modulating host epigenetic regulatory factors, thereby linking cccDNA to hepatocarcinogenesis (**Figure 2**). HBV cccDNA and pgRNA levels represent HBV replication in the liver and might contribute to the progression of HCC in HBsAg carriers and patients with occult HBV infection^3,81,82^. Many factors may modulate cccDNA minichromosome behavior and drive hepatocarcinogenesis. The HBx-mediated biological control of the HBV cccDNA minichromosome is closely associated with the development of HBV-related HCC *via* host-virus interaction. HBx-activated Notch signaling may play an important role not only in HBV-related HCC but also in facilitating HBV cccDNA transcription *via* CREB and subsequent triggering of the downstream PKA-phospho-CREB cascade^67,83^. Our group has reported that HBx-elevated MSL2 regulates the HBV cccDNA minichromosome in hepatoma cells, thus promoting the development of HCC and forming a positive feedback loop of HBx/MSL2/cccDNA/HBV^55^. Interestingly, antiviral therapy modulates hepatocarcinogenesis by decreasing the levels of HBx and inhibiting the tumorigenic effects of MSL2 and the cccDNA minichromosome^84^.

**Figure 3:**
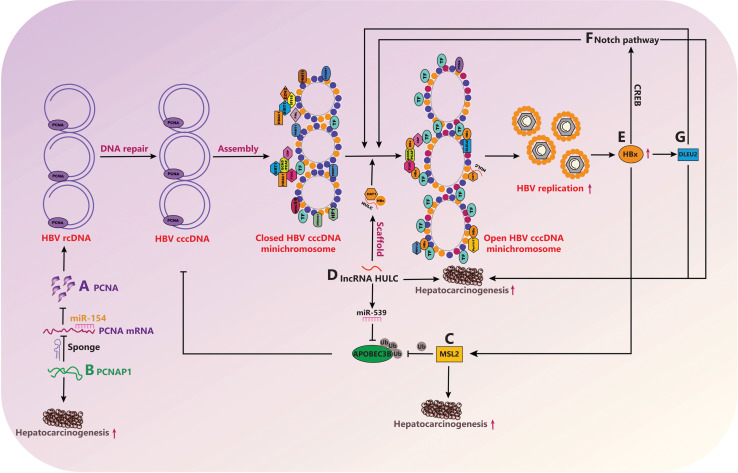
HBV cccDNA and HCC. (A, B) PCNAP1 enhances HBV replication through modulating miR-154/PCNA/HBV cccDNA signaling. PCNAP1/PCNA signaling drives hepatocarcinogenesis. (C) HBx-elevated MSL2 modulates HBV cccDNA in hepatoma cells, thus promoting the development of HCC, forming a positive feedback loop of HBx/MSL2/cccDNA/HBV. (D) HULC enhances HBV cccDNA minichromosome stability by down-regulating APOBEC3B in hepatoma cells, thus mediating the growth of hepatoma cells. (E, F) HBx activating Notch signaling has an important role in HBV-related HCC and facilitates cccDNA transcription *via* CREB. (G) Co-recruitment of the lncRNA DLEU2 and HBx to cccDNA displaces EZH2 from the viral chromatin, and boosts cccDNA transcription and HBV replication, which is associated with the development of HCC.

LncRNAs are regulators involved in biological processes, and their functional disruption has been implicated in the etiology of HCC^85^. Our group has reported that the lncRNA PCNAP1 enhances HBV replication through modulating miR-154/PCNA/HBV cccDNA signaling, in which PCNAP1/PCNA signaling drives hepatocarcinogenesis^39^. In addition, the lncRNA HULC activates HBV by modulating HBx/STAT3/miR-539/APOBEC3B signaling in HBV-related HCC. In brief, HULC enhances HBV cccDNA minichromosome stability by down-regulating APOBEC3B in hepatoma cells, thus leading to the growth of hepatoma cells by activation of HBV *in vitro* and *in vivo*^56^. Furthermore, computational modeling and biochemical evidence suggest that co-recruitment of the lncRNA DLEU2 and HBx to cccDNA displaces EZH2 from viral chromatin, thus boosting transcription and viral replication in HBV-infected cells and HBV-related HCCs^37^. The above findings are summarized in **Figure 3**. In this model, HBx up-regulates and recruits host factors to cccDNA, thereby enhancing the transcription of cccDNA by epigenetic regulation and leading to the replication of HBV through a positive feedback loop. Meanwhile, HBx up-regulates host factors that enhance the growth of liver cancer or directly result in the development of hepatocarcinogenesis.

## Detection of HBV cccDNA

Drug development targeting cccDNA has been hindered by a lack of reliable cccDNA detection methods. Southern blot analysis is regarded as the “gold standard” for quantitative cccDNA detection and remains a widely accepted method^86^. qPCR technology is another method for cccDNA detection in laboratory settings. To date, several methods associated with qPCR have been established, such as semi-nested and nested qPCR, rolling circle amplification qPCR, and magnetic capture hybridization qPCR^87–92^. In addition, the droplet digital PCR-based cccDNA detection system is a sensitive and accurate method for quantifying cccDNA in HBV-transfected HepG2.2.15 cellular and anti-HBc-positive liver donor samples^93,94^. HBV cccDNA-selective droplet digital PCR is also sensitive in detecting cccDNA and may be a promising strategy for HBV-induced HCC surveillance and antiviral therapy evaluation^95^. Recently, a novel cccDNA quantification assay, cccDNA inversion quantitative PCR (cinqPCR), has been established, in which restriction enzymes are used to invert a DNA sequence close to the gap region of genotype D HBV strains^96^. In addition, Zhang et al. have established a highly sensitive and specific in situ hybridization assay for the detection of cccDNA in liver biopsies from patients with chronic hepatitis B (CHB), which can be used to specifically visualize the localization of cccDNA^6,97^.

However, in clinical settings, given the need for liver biopsy, detecting cccDNA in the liver of HBV-infected patients and quantifying cccDNA fluctuation during antiviral therapy through technologies such as Southern blot analysis are difficult. Currently, serum HBV DNA and HBsAg are the most widely used cccDNA markers to diagnose HBV infection and monitor antiviral therapy^98,99^. Nucleos(t)ide analogue (NA) therapy can decrease serum HBV DNA to undetectable levels, but not the levels of HBsAg and hepatitis B core-related antigen (HBcrAg), or serum HBV RNA from cccDNA. Thus, serum HBV RNA, HBsAg, and HBcrAg appear to be better surrogate markers for cccDNA than serum HBV DNA^100^. Accordingly, serum HBV RNA can serve as a biomarker to predict the natural history of disease in patients with CHB when liver biopsies are unavailable^101^. The measurement of HBV RNA before PEG-IFN based therapy has positive predictive value for maintained virological responses^102,103^. HBV RNA is a sensitive biomarker of continued transcription of cccDNA in HBeAg-negative patients, despite marked HBV DNA suppression by NAs^104^. However, no significant correlation exists between serum HBV RNA and cccDNA copy numbers^98^. Moreover, serum HBV RNA derived from pgRNA in virus-like particles is superior in reflecting the activity of intrahepatic cccDNA in patients with CHB who are receiving NA therapy^105^ or in treatment-naive HBV-infected individuals^106–108^. Recent studies have reported that serum HBV RNA comprises heterogeneous lengths and products of incomplete reverse transcription during viral replication. Thus, the composition of HBV RNA might serve as a biomarker of cccDNA^109,110^.

Recently, a growing body of research has indicated that HBcrAg may serve as a new serum biomarker for HBV infection, treatment, and prognosis. HBcrAg contains 3 viral proteins: HBcAg, HBeAg, and a 22 kDa precore protein (p22cr)^111–113^. A correlation between HBcrAg levels and the size of the intrahepatic cccDNA pool has been demonstrated in cohorts of Asian genotype B/C CHB patients^114,115^. Thus, HBcrAg is useful as an HBV re-infection marker after liver transplantation and a marker in HBeAg negative CHB suppressed by NA therapy^104,116^. HBcrAg may serve as a highly sensitive marker reflecting the cccDNA content and persistence of disease even with cccDNA levels below assay detection limits^117^. Furthermore, serum HBcrAg is correlated with cccDNA transcriptional activity in CHB^118^. Nevertheless, whether HBcrAg is better than other factors as a surrogate marker for cccDNA is unclear. Serum HBcrAg is better correlated with cccDNA levels relative to HBV RNA and HBsAg in both HBeAg-positive and HBeAg-negative patients^119^. However, HBcrAg is not superior to HBV DNA and HBsAg in predicting the response during PEG-IFN treatment in white patients with HBeAg-negative CHB^120^.

## IFN and cccDNA

According to cccDNA methods, the development of drug targeting cccDNA is necessary. The current standard therapy for HBV infection includes PEG-IFN-α and NAs. IFN-α may inhibit HBV viral replication through decreasing cccDNA transcription and inducing the degradation of cccDNA. Our group has reported that IFN-α epigenetically regulates the HBV cccDNA minichromosome by modulating GCN5-mediated succinylation of histone H3 lysine 79 (H3K79), thereby suppressing cccDNA transcription^117^. IFN-α inhibits HBV replication by decreasing the transcription of pgRNA and subgenomic RNA through epigenetic regulation of the nuclear cccDNA minichromosome^35^. IFN-α decreases the acetylation levels of histone H3 lysine 9 (H3K9) and 27 (H3K27) in the cccDNA minichromosome, thus inducing long-lasting suppression of cccDNA transcription, and mediates a delayed response that appears to accelerate the decay of cccDNA^121^. Subsequently, the up-regulation of APOBEC3A and APOBEC3B deaminases by IFN-α and lymphotoxin-β receptor agonist have been found to cause partial degradation of cccDNA without hepatotoxicity in HBV-infected cells, primary human hepatocytes, and human liver needle biopsies^1^. An important IFN-α inducible effector, MX2, inhibits HBV infection by decreasing cccDNA minichromosome formation through conversion from rcDNA, rather than destabilizing the existing cccDNA minichromosome^57^. Moreover, UBE2L3, which is correlated with the degradation of APOBEC3A, may be involved in IFN-mediated viral suppression. IFN-α markedly inhibits the expression of UBE2L3 and consequently HBV cccDNA^53^. Three IFN-α-induced cellular proteins, STAT1, SMCHD1, and PML, may be the IFN-α response factors suppressing cccDNA transcription in the silencing of HBV replication^33^. CDM-3008, an interferon-like small chemical compound, suppresses HBV replication and decreases cccDNA levels *via* the activation of the JAK/STAT pathway and induction of interferon-stimulated gene (ISG) expression; the overexpression of ISG15 stimulates HBV production in an ISGylation-dependent manner^122,123^.

**Figure 4:**
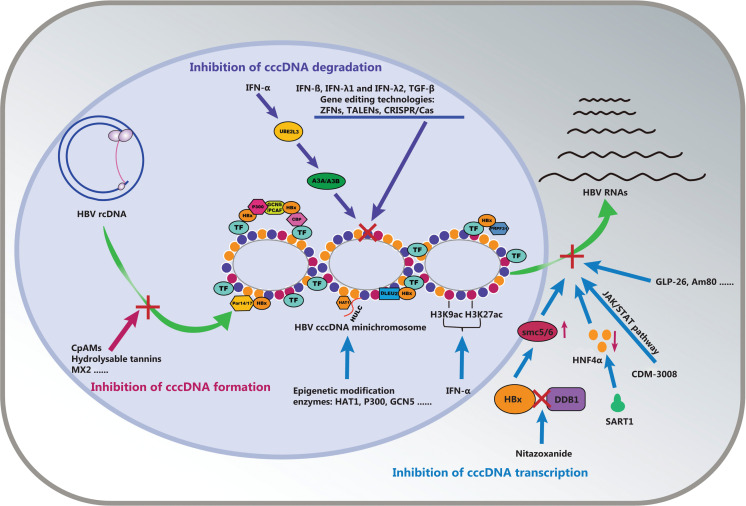
Therapeutic strategies against the cccDNA minichromosome. cccDNA formation: Inhibitors, such as CpAMs, hydrolyzable tannins, and MX2, inhibit cccDNA minichromosome formation through conversion from rcDNA. cccDNA degradation: IFN-α decreases the expression of UBE2L3, which is correlated with the degradation of APOBEC3A, thus decreasing HBV cccDNA. IFN-γ and TNF-α inhibit the levels of HBV cccDNA through up-regulation of APOBEC3A and APOBEC3B deaminases. TGF-β induces cccDNA degradation *via* activation-induced cytidine deaminase (AID) activity. IFN-β, IFN-λ1, IFN-λ2, and gene editing technologies induce the deamination and degradation of cccDNA. cccDNA transcription: epigenetic modification enzyme inhibitors such as HAT1 and p300 inhibitors inhibit the transcription of cccDNA. IFN-α restricts the acetylation levels of histone H3 lysine 9 (H3K9)/27 (H3K27) and the succinylation levels of histone H3 lysine 79 (H3K79) on the cccDNA minichromosome, thereby suppressing cccDNA transcription. Nitazoxanide (NTZ) inhibits HBx-DDB1 protein interaction, thereby accounting for the significant restoration of the SMC5/6 protein level, and suppresses cccDNA transcription and viral protein production. Dicoumarol, an NQO1 inhibitor, blocks cccDNA transcription by promoting the degradation of HBx. CDM-3008, Am80, and IL-6 prevent cccDNA transcription.

IFN-β, IFN-λ1, and IFN-λ2 induce the deamination and degradation of cccDNA, and are similar to IFN-α^124^. IFN-γ and TNF-α inhibit the levels of HBV cccDNA in hepatocytes through up-regulation of APOBEC3A and APOBEC3B deaminases^125^. In addition, TGF-β induces nuclear viral cccDNA degradation and hypermutation *via* activation-induced cytidine deaminase activity in hepatocytes^126^. Functional restoration of CD56^bright^ NK cells in entecavir-treated patients who are switched to PEG-IFN-α contributes to cccDNA clearance through TRAIL-induced cytolysis and TNF-α/IFNγ-mediated noncytolytic pathways^127^. IL-21-based gene and cellular therapies, as valid candidates for the treatment of chronic HBV infections, have potential in removing cccDNA-bearing hepatocytes *via* activated CD8^+^ T cells together with long-term protective memory^128^. IL-6 inhibits HBV transcription by decreasing the binding of essential transcription factors such as HNF1α, HNF4α, and STAT3 to the cccDNA minichromosome, thus leading to the hypo-acetylation of cccDNA cccDNA-bound histone silencing^129^. The above findings are summarized in **Figure 4**.

## Therapeutic strategies against cccDNA

The cccDNA minichromosome plays pivotal role in the persistence of HBV replication and therefore is a crucial target for the treatment and prognosis of HBV-related diseases, offering a possibility for HBV cure with finite therapy through affecting the assembly/formation of the cccDNA minichromosome and the transcription/stability of cccDNA.

### Inhibition of assembly/formation of the cccDNA minichromosome

We presumed that blocking the assembly of the cccDNA minichromosome might be crucial for cleaning cccDNA in clinical settings. However, inhibitors of assembly of the cccDNA minichromosome have not been identified to date. HAT1 is a potential target for controlling the assembly of the cccDNA minichromosome. CCC-0975 and CCC-0346 are specific inhibitors of HBV cccDNA minichromosome formation from rcDNA^130^. HBV core protein allosteric modulators (CpAMs) inhibit the formation of nucleocapsids by disrupting the binding of pgRNA-bound polymerase and a hexamer consisting of 3 core dimers. CpAMs bind the capsid and inhibit the release of rcDNA into the nucleus^131,132^. Interestingly, CpAMs also inhibit cccDNA minichromosome formation during *de novo* HBV infection^133^. Moreover, hydrolyzable tannins significantly restrict cccDNA minichromosome formation and facilitate the degradation of preexisting cccDNA^134^. Therefore, agents controlling the assembly/formation of the cccDNA minichromosome provide a novel strategy for eradicating HBV cccDNA.

### Decreased cccDNA transcription

Importantly, we presumed that HBx might serve as a crucial target to control cccDNA. As expected, nitazoxanide (NTZ) efficiently inhibits the HBx–DDB1 protein interaction, thus accounting for the significant restoration of SMC5/6 protein level, and suppresses viral transcription and viral protein production in the HBV minicircle system and in PHH cells naturally infected with HBV^135^. Recently, dicoumarol, an NQO1 inhibitor, has been demonstrated to have potent anti-HBV activity by promoting the degradation of HBx and blocking cccDNA transcription^136^. Spliceosome associated factor 1 (SART1) restricts the transcription of HBV cccDNA by suppressing the key HBV transcription factor HNF4α in various HBV models^137^. In addition, curcumin inhibits HBV replication through decreasing the acetylation of cccDNA-bound histones, and it may serve as a cccDNA-targeting anti-HBV agent^138^. Am80 correlates with decreased intracellular viral RNA levels, but not cccDNA copy numbers, thus indicating a persistent inhibition of HBV transcription in HepG2-NTCP cells^139^.

### Disrupting cccDNA minichromosome stability

Strikingly, gene therapy targeting cccDNA is a promising technology for curing chronic HBV. Several methods, including zinc finger nucleases, transcription activator-like effector nucleases (TALENs), and the clustered regularly interspaced short palindromic repeat/CRISPR associated (CRISPR/Cas) system, have been engineered to disrupt HBV cccDNA^140–145^. The three gene editing technologies work similarly through targeting cccDNA sequences by using DNA cleaving enzymes, thus silencing cccDNA expression. However, the potential off-target effects and delivery efficiency to HBV infected hepatocytes should be addressed in the approach to eliminate cccDNA. Preclinical experiments have shown that CRISPR-Cas9-based strategies may lead to mutations and deletions that functionally inactivate cccDNA. Approximately 7% of edited DNA has been found to contain in-frame deletions, thus indicating that a single CRISPR target on the HBV genome may not inactivate cccDNA. Therefore, multiple single guide RNAs targeting different loci on the HBV genome might be required to inactivate cccDNA^131,146^. Lutgehetmann et al.^145^ have evaluated the anti-HBV activity of 4 orthologous CRISPR/Cas9 systems: *Streptococcus pyogenes* (SpCas9), *Streptococcus thermophilus* (StCas9), Cas9 orthologues from *Neisseria meningitidis* (NmCas9), and *Francisella novicida* (FnCas9). Interestingly, SpCas9 and StCas9 effectively target HBV cccDNA for degradation, thereby suppressing HBV replication. StCas9 has been found to be the safest and most effective orthologous CRISPR/Cas9 for targeting HBV. The death of infected cells is a major route for the elimination of cccDNA^147^.

### Indirect therapeutic strategies against cccDNA

RNA interference-based anti-HBV therapy affects cccDNA minichromosome formation *via* destabilizing the pgRNA and inhibiting the translation of viral proteins, such as HBx, that are important for cccDNA minichromosome formation^131,148^. The gRNA-miRNA-gRNA ternary cassette combining CRISPR/Cas9 with an RNA interference approach has shown potent activity in destroying HBV cccDNA and blocking HBV replication^144^. Furthermore, hepadnavirus-infected hepatocytes proliferation induce cccDNA dilution among daughter cells and intrahepatic cccDNA loss^145^. Moreover, NAs, as entry inhibitors, may also play roles in the regulation of cccDNA through affecting the replication of HBV^131^. HBV-specific T cells inhibit HBV replication and decrease cccDNA in infected cells; moreover, direct contact is not required for cytolysis, owing to the secretion of IFN-γ and TNF-α, thus potentially supporting HBV cure approaches^121^. In addition, animal models are crucial in the development of anti-HBV drugs. A human chimeric liver mouse model is available to evaluate the efficacy of antiviral agents targeting the HBV replication cycle^148^ and particularly to study the cccDNA in the liver. The above findings are summarized in **Figure 4**.

## Perspectives

On the basis of mapping the cccDNA minichromosome, we provide new insights into the mechanisms through which host factors modulate the cccDNA minichromosome as well as potential treatment strategies targeting cccDNA. With the perspectives on this topic, we present suggestions for future studies on cccDNA (**Figure 4**), such as the many epigenetic modifying enzymes that suppress cccDNA function. Inhibitors targeting those enzymes are available to eliminate cccDNA. Combined therapy with anti-HBV drugs, such as IFN-α with inhibitors of epigenetic modification enzymes, might potentially have enhanced effectiveness in silencing or eliminating cccDNA, as well as in curing HBV-related diseases. Notably, the development of novel drugs targeting HBx will benefit the therapy of cccDNA and HBV-related cancer.

## References

[1] Lucifora J, Xia Y, Reisinger F, Zhang K, Stadler D, Cheng X (2014). Specific and nonhepatotoxic degradation of nuclear hepatitis B virus cccDNA. Science.

[2] Koniger C, Wingert I, Marsmann M, Rosler C, Beck J, Nassal M (2014). Involvement of the host DNA-repair enzyme TDP2 in formation of the covalently closed circular DNA persistence reservoir of hepatitis B viruses. Proc Natl Acad Sci U S A.

[3] Luo X, Huang Y, Chen Y, Tu Z, Hu J, Tavis JE (2016). Association of hepatitis B virus covalently closed circular DNA and human APOBEC3B in hepatitis B virus-related hepatocellular carcinoma. PLoS One.

[4] Hu J, Protzer U, Siddiqui A (2019). Revisiting hepatitis B virus: challenges of curative therapies. J Virol.

[5] Tropberger P, Mercier A, Robinson M, Zhong W, Ganem DE, Holdorf M (2015). Mapping of histone modifications in episomal HBV cccDNA uncovers an unusual chromatin organization amenable to epigenetic manipulation. Proc Natl Acad Sci U S A.

[6] Li X, Zhao J, Yuan Q, Xia N (2017). Detection of HBV covalently closed circular DNA. Viruses.

[7] Gao W, Hu J (2007). Formation of hepatitis B virus covalently closed circular DNA: removal of genome-linked protein. J Virol.

[8] Levrero M, Pollicino T, Petersen J, Belloni L, Raimondo G, Dandri M (2009). Control of cccDNA function in hepatitis B virus infection. J Hepatol.

[9] Levrero M, Zucman-Rossi J (2016). Mechanisms of HBV-induced hepatocellular carcinoma. J Hepatol.

[10] Schwabe RF, Greten TF (2020). Gut microbiome in HCC–mechanisms, diagnosis and therapy. J Hepatol.

[11] Di Bisceglie AM (2009). Hepatitis B and hepatocellular carcinoma. Hepatology.

[12] Chen Y, Tian Z (2019). HBV-induced immune imbalance in the development of HCC. Front Immunol.

[13] Zhang C, Huang C, Sui X, Zhong X, Yang W, Hu X (2019). Association between gene methylation and HBV infection in hepatocellular carcinoma: a meta-analysis. J Cancer.

[14] Xu C, Zhou W, Wang Y, Qiao L (2014). Hepatitis B virus-induced hepatocellular carcinoma. Cancer Lett.

[15] Jia L, Gao Y, He Y, Hooper JD, Yang P (2020). HBV induced hepatocellular carcinoma and related potential immunotherapy. Pharmacol Res.

[16] Zhang WY, Cai N, Ye LH, Zhang XD (2009). Transformation of human liver L-O2 cells mediated by stable HBx transfection. Acta Pharmacol Sin.

[17] Zhang X, You X, Li N, Zhang W, Gagos S, Wang Q (2012). Involvement of hepatitis B virus X gene (HBx) integration in hepatocarcinogenesis via a recombination of HBx/Alu core sequence/subtelomeric DNA. FEBS Lett.

[18] Wang H, Liu Y, Wang D, Xu Y, Dong R, Yang Y (2019). The upstream pathway of mTOR-mediated autophagy in liver diseases. Cells.

[19] Lu X, Paliogiannis P, Calvisi DF, Chen X (2021). Role of the mammalian target of rapamycin pathway in liver cancer: From molecular genetics to targeted therapies. Hepatology.

[20] Zheng YL, Li L, Jia YX, Zhang BZ, Li JC, Zhu YH (2019). LINC01554-mediated glucose metabolism reprogramming suppresses tumorigenicity in hepatocellular carcinoma *via* downregulating PKM2 expression and inhibiting Akt/mTOR signaling pathway. Theranostics.

[21] Allweiss L, Dandri M (2017). The role of cccDNA in HBV maintenance. Viruses.

[22] Newbold JE, Xin H, Tencza M, Sherman G, Dean J, Bowden S (1995). The covalently closed duplex form of the hepadnavirus genome exists in situ as a heterogeneous population of viral minichromosomes. J Virol.

[23] Bock CT, Schranz P, Schroder CH, Zentgraf H (1994). Hepatitis B virus genome is organized into nucleosomes in the nucleus of the infected cell. Virus Genes.

[24] Bock CT, Schwinn S, Locarnini S, Fyfe J, Manns MP, Trautwein C (2001). Structural organization of the hepatitis B virus minichromosome. J Mol Biol.

[25] Belloni L, Pollicino T, De Nicola F, Guerrieri F, Raffa G, Fanciulli M (2009). Nuclear HBx binds the HBV minichromosome and modifies the epigenetic regulation of cccDNA function. Proc Natl Acad Sci U S A.

[26] Lucifora J, Arzberger S, Durantel D, Belloni L, Strubin M, Levrero M (2011). Hepatitis B virus X protein is essential to initiate and maintain virus replication after infection. J Hepatol.

[27] Lee H, Jeong H, Lee SY, Kim SS, Jang KL (2019). Hepatitis B virus X protein stimulates virus replication *via* DNA methylation of the C-1619 in covalently closed circular DNA. Mol Cells.

[28] Chong CK, Cheng CYS, Tsoi SYJ, Huang FY, Liu F, Fung J (2020). HBV X protein mutations affect HBV transcription and association of histone-modifying enzymes with covalently closed circular DNA. Sci Rep.

[29] Saeed U, Kim J, Piracha ZZ, Kwon H, Jung J, Chwae YJ (2019). Parvulin 14 and parvulin 17 bind to HBx and cccDNA and upregulate hepatitis B virus replication from cccDNA to virion in an HBx-dependent manner. J Virol.

[30] Kinoshita W, Ogura N, Watashi K, Wakita T (2017). Host factor PRPF31 is involved in cccDNA production in HBV-replicating cells. Biochem Biophys Res Commun.

[31] Decorsiere A, Mueller H, van Breugel PC, Abdul F, Gerossier L, Beran RK (2016). Hepatitis B virus X protein identifies the Smc5/6 complex as a host restriction factor. Nature.

[32] Riviere L, Gerossier L, Ducroux A, Dion S, Deng Q, Michel ML (2015). HBx relieves chromatin-mediated transcriptional repression of hepatitis B viral cccDNA involving SETDB1 histone methyltransferase. J Hepatol.

[33] Cheng J, Zhao Q, Zhou Y, Tang L, Sheraz M, Chang J (2020). Interferon alpha induces multiple cellular proteins that coordinately suppress hepadnaviral covalently closed circular DNA transcription. J Virol.

[34] Yang Y, Zhao X, Wang Z, Shu W, Li L, Li Y (2020). Nuclear sensor interferon-inducible protein 16 inhibits the function of hepatitis B virus covalently closed circular DNA by integrating innate immune activation and epigenetic suppression. Hepatology.

[35] Belloni L, Allweiss L, Guerrieri F, Pediconi N, Volz T, Pollicino T (2012). IFN-alpha inhibits HBV transcription and replication in cell culture and in humanized mice by targeting the epigenetic regulation of the nuclear cccDNA minichromosome. J Clin Invest.

[36] Benhenda S, Ducroux A, Riviere L, Sobhian B, Ward MD, Dion S (2013). Methyltransferase PRMT1 is a binding partner of HBx and a negative regulator of hepatitis B virus transcription. J Virol.

[37] Salerno D, Chiodo L, Alfano V, Floriot O, Cottone G, Paturel A (2020). Hepatitis B protein HBx binds the DLEU2 lncRNA to sustain cccDNA and host cancer-related gene transcription. Gut.

[38] Yang G, Feng J, Liu Y, Zhao M, Yuan Y, Yuan H (2019). HAT1 signaling confers to assembly and epigenetic regulation of HBV cccDNA minichromosome. Theranostics.

[39] Feng J, Yang G, Liu Y, Gao Y, Zhao M, Bu Y (2019). LncRNA PCNAP1 modulates hepatitis B virus replication and enhances tumor growth of liver cancer. Theranostics.

[40] Hong X, Kim ES, Guo H (2017). Epigenetic regulation of hepatitis B virus covalently closed circular DNA: Implications for epigenetic therapy against chronic hepatitis B. Hepatology.

[41] Liu K, Ludgate L, Yuan Z, Hu J (2015). Regulation of multiple stages of hepadnavirus replication by the carboxyl-terminal domain of viral core protein in trans. J Virol.

[42] Cui X, Luckenbaugh L, Bruss V, Hu J (2015). Alteration of mature nucleocapsid and enhancement of covalently closed circular DNA formation by hepatitis B virus core mutants defective in complete-virion formation. J Virol.

[43] Guo H, Mao R, Block TM, Guo JT (2010). Production and function of the cytoplasmic deproteinized relaxed circular DNA of hepadnaviruses. J Virol.

[44] Luo J, Luckenbaugh L, Hu H, Yan Z, Gao L, Hu J (2020). Involvement of host ATR-CHK1 pathway in hepatitis B virus covalently closed circular DNA formation. mBio.

[45] Wing PA, Davenne T, Wettengel J, Lai AG, Zhuang X, Chakraborty A (2019). A dual role for SAMHD1 in regulating HBV cccDNA and RT-dependent particle genesis. Life Sci Alliance.

[46] Sheraz M, Cheng J, Tang L, Chang J, Guo JT (2019). Cellular DNA topoisomerases are required for the synthesis of hepatitis B virus covalently closed circular DNA. J Virol.

[47] Kitamura K, Que L, Shimadu M, Koura M, Ishihara Y, Wakae K (2018). Flap endonuclease 1 is involved in cccDNA formation in the hepatitis B virus. PLoS Pathog.

[48] Qi Y, Gao Z, Xu G, Peng B, Liu C, Yan H (2016). DNA Polymerase kappa is a key cellular factor for the formation of covalently closed circular DNA of hepatitis B virus. PLoS Pathog.

[49] Tang L, Sheraz M, McGrane M, Chang J, Guo JT (2019). DNA Polymerase alpha is essential for intracellular amplification of hepatitis B virus covalently closed circular DNA. PLoS Pathog.

[50] Long Q, Yan R, Hu J, Cai D, Mitra B, Kim ES (2017). The role of host DNA ligases in hepadnavirus covalently closed circular DNA formation. PLoS Pathog.

[51] Wei L, Ploss A (2020). Core components of DNA lagging strand synthesis machinery are essential for hepatitis B virus cccDNA formation. Nat Microbiol.

[52] Brezgin S, Kostyusheva A, Bayurova E, Gordeychuk I, Isaguliants M, Goptar I (2019). Replenishment of hepatitis B virus cccDNA pool is restricted by baseline expression of host restriction factors *in vitro*. Microorganisms.

[53] Zhou L, Ren JH, Cheng ST, Xu HM, Chen WX, Chen DP (2019). A Functional variant in ubiquitin conjugating enzyme E2 L3 contributes to hepatitis B virus infection and maintains covalently closed circular DNA stability by inducing degradation of apolipoprotein B mRNA editing enzyme catalytic subunit 3A. Hepatology.

[54] Liu Z, Wang J, Yuan H, Liu L, Bu Y, Zhao M (2020). IFN-alpha2b inhibits the ethanol enriched-HBV cccDNA through blocking a positive feedback loop of HBx/MSL2/cccDNA/HBV/HBx in liver. Biochem Biophys Res Commun.

[55] Gao Y, Feng J, Yang G, Zhang S, Liu Y, Bu Y (2017). Hepatitis B virus X protein-elevated MSL2 modulates hepatitis B virus covalently closed circular DNA by inducing degradation of APOBEC3B to enhance hepatocarcinogenesis. Hepatology.

[56] Liu Y, Feng J, Sun M, Yang G, Yuan H, Wang Y (2019). Long non-coding RNA HULC activates HBV by modulating HBx/STAT3/miR-539/APOBEC3B signaling in HBV-related hepatocellular carcinoma. Cancer Lett.

[57] Wang YX, Niklasch M, Liu T, Wang Y, Shi B, Yuan W (2020). Interferon-inducible MX2 is a host restriction factor of hepatitis B virus replication. J Hepatol.

[58] Flecken T, Meier MA, Skewes-Cox P, Barkan DT, Heim MH, Wieland SF (2019). Mapping the heterogeneity of Histone modifications on hepatitis B virus DNA using liver needle biopsies obtained from chronically infected patients. J Virol.

[59] Ren JH, Hu JL, Cheng ST, Yu HB, Wong VKW, Law BYK (2018). SIRT3 restricts hepatitis B virus transcription and replication through epigenetic regulation of covalently closed circular DNA involving suppressor of variegation 3-9 homolog 1 and SET domain containing 1A histone methyltransferases. Hepatology.

[60] Guerrieri F, Belloni L, D’Andrea D, Pediconi N, Le Pera L, Testoni B (2017). Genome-wide identification of direct HBx genomic targets. BMC Genomics.

[61] Slagle BL, Bouchard MJ (2016). Hepatitis B virus X and regulation of viral gene expression. Cold Spring Harb Perspect Med.

[62] Guo YH, Li YN, Zhao JR, Zhang J, Yan Z (2011). HBc binds to the CpG islands of HBV cccDNA and promotes an epigenetic permissive state. Epigenetics.

[63] Zhang Y, Mao R, Yan R, Cai D, Zhang Y, Zhu H (2014). Transcription of hepatitis B virus covalently closed circular DNA is regulated by CpG methylation during chronic infection. PLoS One.

[64] Jain S, Chang TT, Chen S, Boldbaatar B, Clemens A, Lin SY (2015). Comprehensive DNA methylation analysis of hepatitis B virus genome in infected liver tissues. Sci Rep.

[65] Chong CK, Cheng CYS, Tsoi SYJ, Huang FY, Liu F, Seto WK (2017). Role of hepatitis B core protein in HBV transcription and recruitment of histone acetyltransferases to cccDNA minichromosome. Antiviral Res.

[66] Lucifora J, Pastor F, Charles É, Pons C, Auclair H, Fusil F (2021). Evidence for long-term association of virion-delivered HBV core protein with cccDNA independently of viral protein production. JHEP reports: innovation in hepatology.

[67] Wang Z, Kawaguchi K, Honda M, Hashimoto S, Shirasaki T, Okada H (2019). Notch signaling facilitates hepatitis B virus covalently closed circular DNA transcription via cAMP response element-binding protein with E3 ubiquitin ligase-modulation. Sci Rep.

[68] Zeng J, Wu D, Hu H, Young JAT, Yan Z, Gao L (2020). Activation of the liver X receptor pathway inhibits HBV replication in primary human Hepatocytes. Hepatology.

[69] Yuan Y, Zhao K, Yao Y, Liu C, Chen Y, Li J (2019). HDAC11 restricts HBV replication through epigenetic repression of cccDNA transcription. Antiviral Res.

[70] Zhang W, Chen J, Wu M, Zhang X, Zhang M, Yue L (2017). PRMT5 restricts hepatitis B virus replication through epigenetic repression of covalently closed circular DNA transcription and interference with pregenomic RNA encapsidation. Hepatology.

[71] Sekiba K, Otsuka M, Ohno M, Yamagami M, Kishikawa T, Seimiya T (2019). Pevonedistat, a neuronal precursor cell-expressed developmentally down-regulated protein 8-activating enzyme inhibitor, is a potent inhibitor of hepatitis B virus. Hepatology.

[72] Liu N, Zhang J, Yang X, Jiao T, Zhao X, Li W (2017). HDM2 promotes NEDDylation of hepatitis B virus HBx to enhance its stability and function. J Virol.

[73] Han S, Shin H, Oh JW, Oh YJ, Her NG, Nam DH (2019). The protein neddylation inhibitor MLN4924 suppresses patient-derived glioblastoma cells *via* inhibition of ERK and AKT signaling. Cancers.

[74] Zhang H, Xing Z, Mani SK, Bancel B, Durantel D, Zoulim F (2016). RNA helicase DEAD box protein 5 regulates Polycomb repressive complex 2/Hox transcript antisense intergenic RNA function in hepatitis B virus infection and hepatocarcinogenesis. Hepatology.

[75] Moon IY, Choi JH, Chung JW, Jang ES, Jeong SH, Kim JW (2019). MicroRNA20 induces methylation of hepatitis B virus covalently closed circular DNA in human hepatoma cells. Mol Med Rep.

[76] Xing T, Zhu J, Xian J, Li A, Wang X, Wang W (2019). miRNA-548ah promotes the replication and expression of hepatitis B virus by targeting histone deacetylase 4. Life Sci.

[77] Xia Y, Guo H (2020). Hepatitis B virus cccDNA: Formation, regulation and therapeutic potential. Antiviral Res.

[78] Hamada-Tsutsumi S, Naito Y, Sato S, Takaoka A, Kawashima K, Isogawa M (2019). The antiviral effects of human microRNA miR-302c-3p against hepatitis B virus infection. Aliment Pharmacol Ther.

[79] Bai F, Yano Y, Fukumoto T, Takebe A, Tanaka M, Kuramitsu K (2013). Quantification of pregenomic RNA and covalently closed circular DNA in hepatitis B virus-related hepatocellular carcinoma. Int J Hepatol.

[80] Meng C, Liu T, Liu YW, Zhang LZ, Wang YL (2019). Hepatitis B virus cccDNA in hepatocellular carcinoma tissue increases the risk of recurrence after liver transplantation. Transplant Proc.

[81] Gao J, Xiong Y, Wang Y, Wang Y, Zheng G, Xu H (2016). Hepatitis B virus X protein activates Notch signaling by its effects on Notch1 and Notch4 in human hepatocellular carcinoma. Int J Oncol.

[82] Jin XL, Hong SK, Kim H, Lee SK, Yi NJ, Lee KW (2019). Antiviral therapy may decrease HBx, affecting cccDNA and MSL2 in hepatocarcinogenesis. Oncol Lett.

[83] Moyo B, Nicholson SA, Arbuthnot PB (2016). The role of long non-coding RNAs in hepatitis B virus-related hepatocellular carcinoma. Virus Res.

[84] Cai D, Nie H, Yan R, Guo JT, Block TM, Guo H (2013). A southern blot assay for detection of hepatitis B virus covalently closed circular DNA from cell cultures. Methods Mol Biol.

[85] Mazet-Wagner AA, Baclet MC, Loustaud-Ratti V, Denis F, Alain S (2006). Real-time PCR quantitation of hepatitis B virus total DNA and covalently closed circular DNA in peripheral blood mononuclear cells from hepatitis B virus-infected patients. J Virol Methods.

[86] Xu CH, Li ZS, Dai JY, Zhu HY, Yu JW, Lu SL (2011). Nested real-time quantitative polymerase chain reaction assay for detection of hepatitis B virus covalently closed circular DNA. Chin Med J (Engl).

[87] Takkenberg RB, Zaaijer HL, Menting S, Weegink CJ, Terpstra V, Cornelissen M (2010). Detection of hepatitis B virus covalently closed circular DNA in paraffin-embedded and cryo-preserved liver biopsies of chronic hepatitis B patients. Eur J Gastroenterol Hepatol.

[88] Zhong Y, Hu S, Xu C, Zhao Y, Xu D, Zhao Y (2014). A novel method for detection of HBVcccDNA in hepatocytes using rolling circle amplification combined with in situ PCR. BMC Infect Dis.

[89] Guo Y, Sheng S, Nie B, Tu Z (2015). Development of magnetic capture hybridization and quantitative polymerase chain reaction for hepatitis B virus covalently closed circular DNA. Hepat Mon.

[90] Jiang PX, Mao RC, Dong MH, Yu XP, Xun Q, Wang JY (2019). Exonuclease I and III improve the detection efficacy of hepatitis B virus covalently closed circular DNA. Hepatobiliary Pancreat Dis Int.

[91] Mu D, Yan L, Tang H, Liao Y (2015). A sensitive and accurate quantification method for the detection of hepatitis B virus covalently closed circular DNA by the application of a droplet digital polymerase chain reaction amplification system. Biotechnol Lett.

[92] Caviglia GP, Abate ML, Tandoi F, Ciancio A, Amoroso A, Salizzoni M (2018). Quantitation of HBV cccDNA in anti-HBc-positive liver donors by droplet digital PCR: A new tool to detect occult infection. J Hepatol.

[93] Huang JT, Yang Y, Hu YM, Liu XH, Liao MY, Morgan R (2018). A highly sensitive and robust method for hepatitis B virus covalently closed circular DNA detection in single cells and serum. J Mol Diagn.

[94] Zhang X, Lu W, Zheng Y, Wang W, Bai L, Chen L (2016). In situ analysis of intrahepatic virological events in chronic hepatitis B virus infection. J Clin Invest.

[95] European association for the study of the liver (2017). Electronic address EEE, European Association for the Study of the Liver. EASL 2017 Clinical Practice Guidelines on the management of hepatitis B virus infection. J Hepatol.

[96] Tu T, Zehnder B, Qu B, Ni Y, Main N, Allweiss L (2020). A novel method to precisely quantify hepatitis B virus covalently closed circular (ccc)DNA formation and maintenance. Antiviral Res.

[97] Gao Y, Li Y, Meng Q, Zhang Z, Zhao P, Shang Q (2017). Serum hepatitis B virus DNA, RNA, and HBsAg: Which correlated better with intrahepatic covalently closed circular DNA before and after nucleos(t)ide analogue treatment?. J Clin Microbiol.

[98] Liu Y, Jiang M, Xue J, Yan H, Liang X (2019). Serum HBV RNA quantification: useful for monitoring natural history of chronic hepatitis B infection. BMC Gastroenterol.

[99] Huang Q, Zhou B, Cai D, Zong Y, Wu Y, Liu S (2021). Rapid turnover of hepatitis B virus covalently closed circular DNA Indicated by monitoring emergence and reversion of signature-mutation in treated chronic hepatitis B patients. Hepatology.

[100] Limothai U, Chuaypen N, Poovorawan K, Chotiyaputta W, Tanwandee T, Poovorawan Y (2019). Baseline and kinetics of serum hepatitis B virus RNA predict response to pegylated interferon-based therapy in patients with hepatitis B e antigen-negative chronic hepatitis B. J Viral Hepat.

[101] van Campenhout MJH, van Bommel F, Pfefferkorn M, Fischer J, Deichsel D, Boonstra A (2020). Serum hepatitis B virus RNA predicts response to peginterferon treatment in HBeAg-positive chronic hepatitis B. J Viral Hepat.

[102] Wang J, Yu Y, Li G, Shen C, Meng Z, Zheng J (2018). Relationship between serum HBV-RNA levels and intrahepatic viral as well as histologic activity markers in entecavir-treated patients. J Hepatol.

[103] Wang J, Shen T, Huang X, Kumar GR, Chen X, Zeng Z (2016). Serum hepatitis B virus RNA is encapsidated pregenome RNA that may be associated with persistence of viral infection and rebound. J Hepatol.

[104] Huang H, Wang J, Li W, Chen R, Chen X, Zhang F (2018). Serum HBV DNA plus RNA shows superiority in reflecting the activity of intrahepatic cccDNA in treatment-naive HBV-infected individuals. J Clin Virol.

[105] Bai L, Zhang X, Kozlowski M, Li W, Wu M, Liu J (2018). Extracellular hepatitis B virus RNAs are heterogeneous in length and circulate as capsid-antibody complexes in addition to virions in chronic hepatitis B patients. J Virol.

[106] Shen S, Xie Z, Cai D, Yu X, Zhang H, Kim ES (2020). Biogenesis and molecular characteristics of serum hepatitis B virus RNA. PLoS Pathog.

[107] Kimura T, Rokuhara A, Sakamoto Y, Yagi S, Tanaka E, Kiyosawa K (2002). Sensitive enzyme immunoassay for hepatitis B virus core-related antigens and their correlation to virus load. J Clin Microbiol.

[108] Kimura T, Ohno N, Terada N, Rokuhara A, Matsumoto A, Yagi S (2005). Hepatitis B virus DNA-negative dane particles lack core protein but contain a 22-kDa precore protein without C-terminal arginine-rich domain. J Biol Chem.

[109] Rokuhara A, Tanaka E, Matsumoto A, Kimura T, Yamaura T, Orii K (2003). Clinical evaluation of a new enzyme immunoassay for hepatitis B virus core-related antigen; a marker distinct from viral DNA for monitoring lamivudine treatment. J Viral Hepat.

[110] Chen EQ, Feng S, Wang ML, Liang LB, Zhou LY, Du LY (2017). Serum hepatitis B core-related antigen is a satisfactory surrogate marker of intrahepatic covalently closed circular DNA in chronic hepatitis B. Sci Rep.

[111] Wang L, Cao X, Wang Z, Gao Y, Deng J, Liu X (2019). Correlation of HBcrAg with intrahepatic hepatitis B virus total DNA and covalently closed circular DNA in HBeAg-positive chronic hepatitis B patients. J Clin Microbiol.

[112] Matsuzaki T, Tatsuki I, Otani M, Akiyama M, Ozawa E, Miuma S (2013). Significance of hepatitis B virus core-related antigen and covalently closed circular DNA levels as markers of hepatitis B virus re-infection after liver transplantation. J Gastroenterol Hepatol.

[113] Wong DK, Seto WK, Cheung KS, Chong CK, Huang FY, Fung J (2017). Hepatitis B virus core-related antigen as a surrogate marker for covalently closed circular DNA. Liver Int.

[114] Testoni B, Lebosse F, Scholtes C, Berby F, Miaglia C, Subic M (2019). Serum hepatitis B core-related antigen (HBcrAg) correlates with covalently closed circular DNA transcriptional activity in chronic hepatitis B patients. J Hepatol.

[115] Chen EQ, Wang ML, Tao YC, Wu DB, Liao J, He M (2019). Serum HBcrAg is better than HBV RNA and HBsAg in reflecting intrahepatic covalently closed circular DNA. J Viral Hepat.

[116] van Campenhout MJH, Rijckborst V, Brouwer WP, van Oord GW, Ferenci P, Tabak F (2019). Hepatitis B core-related antigen monitoring during peginterferon alfa treatment for HBeAg-negative chronic hepatitis B. J Viral Hepat.

[117] Yuan Y, Yuan H, Yang G, Yun H, Zhao M, Liu Z (2020). IFN-alpha confers epigenetic regulation of HBV cccDNA minichromosome by modulating GCN5-mediated succinylation of histone H3K79 to clear HBV cccDNA. Clin Epigenetics.

[118] Liu F, Campagna M, Qi Y, Zhao X, Guo F, Xu C (2013). Alpha-interferon suppresses hepadnavirus transcription by altering epigenetic modification of cccDNA minichromosomes. PLoS Pathog.

[119] Furutani Y, Toguchi M, Shiozaki-Sato Y, Qin XY, Ebisui E, Higuchi S (2019). An interferon-like small chemical compound CDM-3008 suppresses hepatitis B virus through induction of interferon-stimulated genes. PLoS One.

[120] Bockmann JH, Stadler D, Xia Y, Ko C, Wettengel JM, Schulze Zur Wiesch J (2019). Comparative analysis of the antiviral effects mediated by type I and III interferons in hepatitis B virus-infected hepatocytes. J Infect Dis.

[121] Xia Y, Stadler D, Lucifora J, Reisinger F, Webb D, Hösel M (2016). Interferon-γ and tumor necrosis factor-α produced by T cells reduce the HBV persistence form, cccDNA, without cytolysis. Gastroenterology.

[122] Qiao Y, Han X, Guan G, Wu N, Sun J, Pak V (2016). TGF-beta triggers HBV cccDNA degradation through AID-dependent deamination. FEBS Lett.

[123] Shi A, Zhang X, Xiao F, Zhu L, Yan W, Han M (2018). CD56(bright) natural killer cells induce HBsAg reduction via cytolysis and cccDNA decay in long-term entecavir-treated patients switching to peginterferon alfa-2a. J Viral Hepat.

[124] Shen Z, Liu J, Wu J, Zhu Y, Li G, Wang J (2019). IL-21-based therapies induce clearance of hepatitis B virus persistence in mouse models. Theranostics.

[125] Palumbo GA, Scisciani C, Pediconi N, Lupacchini L, Alfalate D, Guerrieri F (2015). IL6 inhibits HBV transcription by targeting the epigenetic control of the nuclear cccDNA minichromosome. PLoS One.

[126] Cai D, Mills C, Yu W, Yan R, Aldrich CE, Saputelli JR (2012). Identification of disubstituted sulfonamide compounds as specific inhibitors of hepatitis B virus covalently closed circular DNA formation. Antimicrob Agents Chemother.

[127] Fanning GC, Zoulim F, Hou J, Bertoletti A (2019). Therapeutic strategies for hepatitis B virus infection: towards a cure. Nat Rev Drug Discov.

[128] Amblard F, Boucle S, Bassit L, Cox B, Sari O, Tao S (2020). Novel hepatitis B virus capsid assembly modulator induces potent antiviral responses *in vitro* and in humanized mice. Antimicrob Agents Chemother.

[129] Ko C, Bester R, Zhou X, Xu Z, Blossey C, Sacherl J (2019). A new role for capsid assembly modulators to target mature hepatitis B virus capsids and prevent virus infection. Antimicrob Agents Chemother.

[130] Liu C, Cai D, Zhang L, Tang W, Yan R, Guo H (2016). Identification of hydrolyzable tannins (punicalagin, punicalin and geraniin) as novel inhibitors of hepatitis B virus covalently closed circular DNA. Antiviral Res.

[131] Sekiba K, Otsuka M, Ohno M, Yamagami M, Kishikawa T, Suzuki T (2019). Inhibition of HBV transcription from cccDNA with nitazoxanide by targeting the HBx-DDB1 interaction. Cell Mol Gastroenterol Hepatol.

[132] Cheng ST, Hu JL, Ren JH, Yu HB, Zhong S, Wai Wong VK (2021). Dicoumarol, an NQO1 inhibitor, blocks cccDNA transcription by promoting degradation of HBx. J Hepatol.

[133] Wei ZQ, Zhang YH, Ke CZ, Chen HX, Ren P, He YL (2017). Curcumin inhibits hepatitis B virus infection by down-regulating cccDNA-bound histone acetylation. World J Gastroenterol.

[134] Nkongolo S, Nussbaum L, Lempp FA, Wodrich H, Urban S, Ni Y (2019). The retinoic acid receptor (RAR) alpha-specific agonist Am80 (tamibarotene) and other RAR agonists potently inhibit hepatitis B virus transcription from cccDNA. Antiviral Res.

[135] Cradick TJ, Keck K, Bradshaw S, Jamieson AC, McCaffrey AP (2010). Zinc-finger nucleases as a novel therapeutic strategy for targeting hepatitis B virus DNAs. Mol Ther.

[136] Schiffer JT, Swan DA, Stone D, Jerome KR (2013). Predictors of hepatitis B cure using gene therapy to deliver DNA cleavage enzymes: a mathematical modeling approach. PLoS Comput Biol.

[137] Teng Y, Xu Z, Zhao K, Zhong Y, Wang J, Zhao L (2021). Novel function of SART1 in HNF4α transcriptional regulation contributes to its antiviral role during HBV infection. J Hepatol.

[138] Kennedy EM, Bassit LC, Mueller H, Kornepati AVR, Bogerd HP, Nie T (2015). Suppression of hepatitis B virus DNA accumulation in chronically infected cells using a bacterial CRISPR/Cas RNA-guided DNA endonuclease. Virology.

[139] Schiwon M, Ehrke-Schulz E, Oswald A, Bergmann T, Michler T, Protzer U (2018). One-vector system for multiplexed CRISPR/Cas9 against hepatitis B virus cccDNA utilizing high-capacity adenoviral vectors. Mol Ther Nucleic Acids.

[140] Kostyushev D, Brezgin S, Kostyusheva A, Zarifyan D, Goptar I, Chulanov V (2019). Orthologous CRISPR/Cas9 systems for specific and efficient degradation of covalently closed circular DNA of hepatitis B virus. Cell Mol Life Sci.

[141] Seeger C, Sohn JA (2016). Complete spectrum of CRISPR/Cas9-induced mutations on HBV cccDNA. Mol Ther.

[142] Zhu Y, Yamamoto T, Cullen J, Saputelli J, Aldrich CE, Miller DS (2001). Kinetics of hepadnavirus loss from the liver during inhibition of viral DNA synthesis. J Virol.

[143] van den Berg F, Limani SW, Mnyandu N, Maepa MB, Ely A, Arbuthnot P (2020). Advances with RNAi-based therapy for hepatitis B virus infection. Viruses.

[144] Wang J, Chen R, Zhang R, Ding S, Zhang T, Yuan Q (2017). The gRNA-miRNA-gRNA ternary cassette combining CRISPR/Cas9 with RNAi approach strongly inhibits hepatitis B virus replication. Theranostics.

[145] Lutgehetmann M, Volz T, Kopke A, Broja T, Tigges E, Lohse AW (2010). In vivo proliferation of hepadnavirus-infected hepatocytes induces loss of covalently closed circular DNA in mice. Hepatology.

[146] Allweiss L, Volz T, Giersch K, Kah J, Raffa G, Petersen J (2018). Proliferation of primary human hepatocytes and prevention of hepatitis B virus reinfection efficiently deplete nuclear cccDNA in vivo. Gut.

[147] Petersen J, Thompson AJ, Levrero M (2016). Aiming for cure in HBV and HDV infection. J Hepatol.

[148] Dandri M, Lutgehetmann M (2014). Mouse models of hepatitis B and delta virus infection. J Immunol Methods.

